# Switching from injectable to other Disease Modifying Therapies may improve sexual dysfunction in people with Multiple Sclerosis

**DOI:** 10.1186/s12883-024-03765-2

**Published:** 2024-07-24

**Authors:** Sara Ala, Ali Amirkafi, Kosar Kohandel, Sareh Shahmohammadi, Mohammad Ali Sahraian

**Affiliations:** 1 Multiple Sclerosis Research Center,Neuroscience Institute, Tehran University of Medical Sciences, Tehran, Iran; 2 Iran University of Medical Sciences, Tehran, Iran

**Keywords:** Multiple sclerosis, Sexual function, Sexual dysfunction, Disease modifying therapies

## Abstract

**Background:**

Multiple Sclerosis (MS) a central nervous system autoimmune disorder, mainly affecting young adults and more prevalent among women, can lead to sexual dysfunction (SD) among both males and females with MS. Female sexual dysfunction can be defined as dyspareunia, a lack of sexual desire, disorders in the arousal and orgasm phases, and sexual pain disorders. The purpose of this study is to investigate the changes in sexual function among females with MS whose treatment was switched from first-line injectable medications to other agents after a six-month duration. And assess the changes in all three domains of SD.

**Methods:**

In this longitudinal study females diagnosed with MS, aged between 18 and 50 years old, and were candidates for switching their treatment from interferon beta-1a (intra-muscular and subcutaneous), and Glatiramer Acetate (GA), to Fingolimod, Dimethyl Fumarate (DMF), or Natalizumab (NTZ) due to patients’ convenience and tolerability and adverse events were included. “Multiple Sclerosis Intimacy and Sexuality Questionnaire-19” was used to evaluate the SD changes before and six months after the new treatment initiation. Statistical analysis was conducted using SPSS V.24 software. Histograms and the Shapiro-Wilk test were used to assess the normality of the variables; due to the non-normal distribution of quantitative variables (except for age), the Wilcoxon signed-rank test was used to compare the scores, before and six months after the medication change. The level of significance was considered less than 0.05.

**Results:**

Out of 107 female participants (average age: 35.09 ± 5.61), The mean of overall MSISQ-19 scores, before and six months after the medication change were not significant (p-value = 0.091). However, considering the subdomains, the medication changes only affected the tertiary subdomain of MSISQ-19 (p-value = 0.017). Still, the scores of other subdomains did not change significantly (p-value = 0.761 for primary SD and 0.479 for secondary SD). Also, there wasn’t any significant difference between EDSS before and after the medication change (p-value = 0.461).

**Conclusions:**

To our knowledge, this was the first study, assessing the effect of MS medication change on the improvement of SD among patients. According to the results of the presented cross-sectional study, we found that during a six-month period, the tertiary subdomain of MSISQ-19 symptoms improved significantly, while the changes in other SD domains were not significant.

## Background

Multiple Sclerosis (MS) is an autoimmune degenerative disorder characterized by the destruction of myelin sheets in the Central Nervous System (CNS) [1]. While being a major cause of disability, it primarily affects young adults and has a higher prevalence among women [2]. In 2020, the number of individuals living with MS was estimated to be 2.8 million worldwide [3]. MS affects patients’ physical health by causing disabilities, easy fatigability, and mental health through depression and anxiety [4]. These factors collectively have a negative impact on the Quality of Life (QoL) and sexual performance, resulting in sexual dysfunction (SD) among patients [5], which limits the sexual satisfaction experience [6].

Contributing factors to SD among individuals with MS can be characterized into three main groups; primary SD (due to demyelinating lesions in the CNS which are responsible for sexual function), secondary SD (due to the impact of neurological symptoms and physical disabilities), tertiary SD (due to emotional and psychosocial complications caused by MS) [7]. Female sexual dysfunction can be defined as dyspareunia, a lack of sexual desire, disorders in the arousal and orgasm phases, and sexual pain disorders [8]; these disorders affect sexual function, leading to reduced satisfaction, and as a result, it affects the mental and social health of women [9]. In addition, SD is usually underdiagnosed and under-treated due to social pressure, taboos, and a lack of specific training for treating physicians [7, 10].

SD is a common complication experienced by individuals with MS, with reported prevalence rates of 55% among women and 62.9% among men [11, 12]. While both men and women with MS can experience SD, symptoms’ manifestations differ between the genders. Men commonly experience erectile dysfunction, while women frequently face decreased libido and orgasm disorders [13, 14].

In addition to the pathology of the disease itself, the origin of SD can be secondary to the side effects of medications used in MS treatment, as well as the medications aimed at reducing the common symptoms among people with MS; such as selective serotonin reuptake inhibitors (SSRIs) to treat depression.

In general, shifting to oral medications can increase the patients’ QoL; as mentioned in similar studies, six months after switching to oral medications, patients have experienced better QoL and fewer complications [15, 16].

While oral and second-line medications sound promising in improving the QoL of patients and various studies have been conducted on the effect and side effects of shifting from injectables to oral and second-line medications, regarding Disease-Modifying Therapies (DMTs) influence on sexual function, there are either insufficient or controversial data. As a first step toward improving knowledge regarding MSISQ-19 score changes resulting from the type of DMTs, we decided to focus on females exclusively, as they have different symptoms and/or psychological conditions from males; therefore, the purpose of this study is to investigate the changes in sexual function among females with MS whose treatment was switched from first-line injectable medications to other agents after a six-month duration.

## Method

### Participants and design

In this longitudinal study female patients diagnosed with MS, who had the following criteria entered the study: (1) were between 18 and 50 years old, (2) were sexually active, (3) have been under treatment with first-line injectable agents including INF beta-1a (intra-muscular and subcutaneous) (Recigen^®^, Cinnovex^®^), Glatiramer Acetate (GA), and were candidates to switch their treatment to Fingolimod, Dimethyl Fumarate (DMF), or Natalizumab (NTZ) due to patients’ convenience and tolerability and adverse events (including injection site reaction, flu-like syndrome, and abnormal Liver Function Tests). The necessary criteria including varicella zoster virus antibody levels and patient lifestyle, were taken into consideration by neurologists before selecting the subsequent DMT. Worth mentioning that considering the cultural issues all participants were married. Patients who have been diagnosed with major depression, diabetes, hypertension, hypothyroidism, and hyperthyroidism and those who are smokers or alcohol consumers were excluded. After giving all the information regarding the aim of the study, procedure, and obtaining written informed consent, the participants were asked to fill out the questionnaire in two phases, before switching the medications and 6 months after the new treatment initiation. Those who could not continue their DMF due to gastrointestinal side effects were excluded from the study. Moreover, we did not enter patients who experienced relapse or MRI activity before switching their DMT in the study. This study is approved by the Tehran University of Medical Sciences Ethical Committee with the ethical code; IR.TUMS.SINAHOSPITAL.REC.1401.125. All the stages of the presented study have been done according to the guidelines and regulations of the research committee of Tehran University of Medical Sciences and researchers followed all steps provided in the primary proposal presented to the committee; and the study was conducted based on the Helsinki declaration.

### Questionnaire

The questionnaire consisted of two parts. The first part was a researcher-made questionnaire to gather demographic data and past medical history; these included: age, education, employment status, and the Expanded Disability Status Scale (EDSS) [17] score at the start of the study and six months after which was determined by a neurologist through neurological examination.

The second part was the “Multiple Sclerosis Intimacy and Sexuality Questionnaire-19” (MSISQ-19) [18]; which is a 19-item questionnaire, assessing sexual problems among females diagnosed with MS; and is classified into three categories: primary dysfunction, consisting of five questions; assessing orgasms, sexual desire, sensory disturbances, and vaginal dryness; secondary dysfunction with nine questions; assessing physical aspects such as bowel and bladder symptoms, tremor, pain or spasticity; and tertiary dysfunction, consisted of five questions; assessing fear of rejection and partner satisfaction, feeling less confidence and less feminine. One main reason for including only females in our study is that the MSIS-19 questionnaire has been validated in Persian exclusively for women.

The scoring was based on a scale ranging from 1 = “never” (never interfered with my sexual activity or satisfaction), 2 = “almost never”, 3 = “occasionally”, 4 = “almost always”, to 5 = “always”. Therefore, it provides an overall score and three subscales for each patient; higher scores imply higher levels of sexual dissatisfaction.

### Statistical analysis

Statistical analysis was conducted using SPSS V.24 software. Quantitative descriptive statistics are expressed as median and IQR (Inter-Quartile Range); qualitative statistics are expressed as numbers and percentages. Histograms and Shapiro-Wilk test was used to assess the normality of the variables; due to the non-normal distribution of quantitative variables (except for age), Wilcoxon signed rank test was used to compare the scores, before and six months after the medication change. Level of significancy was considered less than 0.05.

## Results

### Demographic data

During the study period, 107 participants entered the study. They were all female, with a mean age of 35.09 ± 5.61. Most of them (63.6%) had bachelor’s degrees and were mainly employed (58.9%). The main first line Injectable medication was INF beta-1a IM, once per week (Cinnovex^®^) and the main subsequent treatment was DMF. The mean EDSS score before the medication change was 1.18 ± 0.96 and six months after was 1.11 ± 1.18. Complete data is presented in Table 1.

**Table 1:** Demographic, medical history, and EDSS score of participants

Variable	Number (percentage)	Median (IQR*)
Age		35 (31–40)
Education		
Diploma or less	17 (15.9%)	
Bachelor	68 (63.6%)	
Master	18 (16.8%)	
Doctorate	8 (3.7%)	
Employment		
Employed	63 (58.9%)	
Unemployed	44 (41.1%)	
first-line Injectable agents		
Interferon beta-1a IM	46 (43%)	
Glatiramer Acetate	36 (33.6%)	
Interferon beta-1a SC	25 (23.4%)	
Switched agents		
Dimethyl Fumarate	80 (74.8%)	
Natalizumab	19 (17.8%)	
Fingolimod	8 (7.5%)	
EDSS score before the medication change		1 (0–2)
EDSS score six months after the medication change		1 (0–2)

* Inter-Quartile Range

(p-value = 0.142)

### Comparison of MSISQ-19 scores

The means of overall MSISQ-19 scores, before and six months after the medication change were not significant (p-value = 0.091). However, considering the subdomains, the medication changes only affected the tertiary subdomain of MSISQ-19 (p-value = 0.017); but the scores of other subdomains did not change significantly (p-value = 0.761 for primary SD and 0.479 for secondary SD). Tables 2 and 3, show the Median and IQR and the SD changes before and after the medication change. Also, there wasn’t any significant difference between EDSS before and after the medication change (p-value = 0.461), which is probably because the participants did not experience any disease or MRI activity.

**Table 2:** MSISQ-19 scores, before and six months after the medication change

Variable	Before medication change, Median (IQR)*	6 months after medication change, Median (IQR*)
Total score	34 (29–43)	33 (25–43)
Primary SD	10 (6–13)	10 (7–13)
Secondary SD	17 (12–19)	14 (10–21)
Tertiary subdomain of MSISQ-19	9 (7–11)	7 (6–11)

*Inter-Quartile Range

**Table 3:** Comparison between the MSISQ-19 scores before and six months after medication change based on Wilcoxon signed rank test

	Positive Ranks			Negative Ranks			Test Statistics		
	number	Mean Rank	Sum of Ranks	number	Mean Rank	Sum of Ranks	Ties	Z	P-value
Total score (After – Before)	43	46.29	1990.47	56	52.85	2959.6	8	-1.692	0.091
Primary SD (After – Before)	48	46.78	2245.44	48	50.22	2410.56	11	-0.304	0.761
Secondary SD (After – Before)	43	44.52	1914.36	48	47.32	2271.36	16	-0.708	0.479
Tertiary subdomain of MSISQ-19 (After – Before)	29	40.93	1186.97	53	41.81	2215.93	25	-2.387	0.017

## Discussion

According to the results of the presented longitudinal study, changing medications from INF beta-1a and GA to DMF, NTZ, and Fingolimod in females with MS improved their Tertiary subdomain of MSISQ-19.

Tertiary SD(Tertiary subdomain of MSISQ-19) is associated with the psychological, emotional, social, and cultural effects of MS; these include depression, anxiety, misrepresented body image, low self-esteem, fear of becoming dependent on a partner, sexual rejection, pain during intercourse, and shifting roles in family are categorized as tertiary SD (Tertiary subdomain of MSISQ-19) [19]; according to some studies more MS patients have complaints related to the tertiary SD (Tertiary subdomain of MSISQ-19), compared to primary and secondary types [20].

Some studies expressed that secondary SD is more frequent; it was probably because patients had more intestinal symptoms, spasticity, weakness in extremities, burning pain, difficulty concentrating, and cognitive dysfunction [21]. Also, one typical symptom of MS that is linked to secondary SD is severe fatigue; so, even in the early stages of the disease, when substantial disability is absent, sexual disorders can be frequently seen among female MS patients. [22, 23].

The common side effects of the prior medications in our investigation included flu-like symptoms and injection site reactions. On one hand, injection site reactions can cause skin changes which might affect the woman’s body image and lead to lower sexual confidence [24]. On the other hand, The night time of injection, occurrence of flu-like symptoms, and subsequent fatigue in patients who receive interferon drugs may have negative effects on the sexual function [25, 26]. Moreover, according to the literature, INF beta-1a may induce depression or worsen an individual’s depression symptoms. In fact, there is evidence suggestive that treatment with INF beta-1a may increase depression symptoms [27, 28]. Altogether, there is no proven positive effect of interferons on improving sexual function among MS patients [29–31].

In the presented study, the main first and subsequent treatments were interferons and DMF, respectively. One of the most commonly known side effects of Interferons is depression. However, according to the literature, DMF is related to less disturbance in depression, improved adherence scores and treatment quality assessment, and reported significantly better QoL and functioning in comparison with GA and INF beta-1a injection [32–34]. Furthermore, the sustained positive effects of DMF on sleep quality can also indirectly improve the sexual performance of patients compared to interferons [35, 36]. Although all together, there is scarce data regarding each DMT’s specific effects on different domains of sexual functions. Additionally, Scandurra and Carotenuto et al. in their investigations have been shown, negative sickness perception, both directly and indirectly (via depressive symptoms), mediates the effect of objective physical disability on SD in multiple sclerosis, also in other literature, they found disability impressed depression in MS patients during the pandemic period of COVID-19, through the moderating role of exercise and physical activity [37, 38].

INF beta-1a, GA, and DMF are considered as first-line treatments; exhibiting more or less comparable effectiveness in preventing MRI or disease activity [39]. However, NTZ shows higher efficacy in highly active MS and better control in preventing disease progression [40, 41]. Considering these findings, we expected that if DMF demonstrates similar efficacy in preventing disease activity as previous treatments, the primary and secondary MSISQ-19 scores —related to individuals’ physical and neurological aspects—would remain unchanged. Since in the most of participants, previous medication altered to DMF, on the other hand, primary and secondary domains contribute significantly to the overall MSISQ-19 scores, the MSISQ-19 scores did not show substantial improvement.

According to Robertson et al., patients with established SD reported fewer symptoms with NTZ therapy, as indicated by a decrease in the primary subscale of the MSISQ-19 [42], In addition, some studies found that most subjects who received NTZ for one year experienced improvement or stabilization in their EDSS status [43]. In conclusion, NTZ injection can improve the EDSS score and as discussed before, secondary MSISQ-19 scores are related to EDSS scores. So, NTZ can impress primary and secondary MSISQ-19 scores; Given that a small percentage of patients in our study had switched from injectable medications to NTZ, therefore; Secondary and primary SD significantly, were less affected than the tertiary MSISQ-19 scores.

One reason that explains the insignificant change in primary and secondary MS scores is the follow-up time. Perhaps the most important reason for the non-significance of change in primary and secondary SD is the short follow-up period because the results of improvement of symptoms and better control of the disease activity and its effects on sexual function require more follow-up time. Of course, in the cases of the NTZ treatment, due to its high efficacy and the short time of onset of action of the drug in controlling the disease activity, improving primary SD has been achieved in a short period of 24 weeks [42, 44]. In our findings, over six months of follow-up, the changes of EDSS score were insignificant. The main reason for switching DMTs which was patient convenience can explain the almost consistent EDSS scores over study time.

## Limitations

One limitation of our study is attributed to the design of the study; as we used an observational design, with no control group, it is hard to make a solid conclusion regarding the observed improvement in tertiary MSISQ-19 scores after treatment change. Another limitation is the follow-up time, as all three aspects of SD, particularly primary and secondary SD need longer periods to change. Finally, an equal share of participants in the consumption of each medication can help, comparing the effect of each medication separately.

## Conclusion

Based on our knowledge, this was the first study, assessing the effect of MS medication change on the improvement of SD among patients. We found that during a six-month period, the Tertiary subdomain of MSISQ-19 symptoms improved significantly, while the changes for other SD domains were not significant. Our findings could open a path for future studies to further assess the effect of medication change by longer follow-ups and interventional designs.

We did not examine depression levels, and only relied on the psychological aspects of the tertiary sexual dysfunction domain in the MSISQ-19 questionnaire, in future studies we suggest evaluating the psychological aspects of patients in collaboration with a psychiatrist to determine the probable depression in the study population.

Cultural issues are the main reason for the deprivation of women with MS from sexual consultations and education. Consequently, patients in particular women with MS, mostly do not discuss their sexual disturbances with neurologists. This can have several reasons; for instance, considering sexual talks as taboo, fear of rejection by their partner, and medical staff judgments. Neurologists and nurses, together with patients should receive proper educational training in order to overcome these barriers.

## Data Availability

The datasets used and/or analyzed during the current study are available from the corresponding author on reasonable request.

## Abbreviations

MS: Multiple Sclerosis
CNS: Central Nervous System
SD: Sexual Dysfunction
DMTs: Disease Modifying Therapies
QoL: Quality of Life
DMF: Dimethyl Fumarate
NTZ: Natalizumab
GA: Glatiramer Acetate
INF bata-1a: Interferon beta-1a
EDSS: Extended Disability Status Scale
